# Combination Radioimmunotherapy Strategies for Solid Tumors

**DOI:** 10.3390/ijms20225579

**Published:** 2019-11-08

**Authors:** Javeria Zaheer, Hyeongi Kim, Yong-Jin Lee, Jin Su Kim, Sang Moo Lim

**Affiliations:** 1 Division of RI application, Korea Institute of Radiological and Medical Sciences, (KIRAMS), 75 Nowon-ro, Nowon-gu, Seoul 01812, Korea; javeria24@kirams.re.kr (J.Z.); erwin.hyeongi@gmail.com (H.K.); yjlee@kirams.re.kr (Y.-J.L.); smlim328@kirams.re.kr (S.M.L.); 2 Radiological and Medico-Oncological Sciences, University of Science and Technology (UST), 75 Nowon-ro, Nowon-gu, Seoul 01812, Korea

**Keywords:** radioimmunotherapy, solid tumors, monoclonal antibody, extracellular matrix, cell-to-cell junctions, interstitial pressure, immune suppressive environment

## Abstract

Combination radioimmunotherapy is an emerging approach for the treatment of solid tumors where radio immunotherapy alone has proven to be reasonably ineffective. Radioimmunotherapy (RIT) using monoclonal antibodies (mAbs) labeled with radionuclides is an attractive approach for cancer treatment because tumor-associated mAbs with cytotoxic radionuclides can selectively bind to tumor antigens. However, due to various limitations, mAbs cannot reach solid tumors, consequently reducing RIT efficacy. Combination RIT is a pragmatic approach through which the addition of drugs or other agents not only help mAbs to reach the targeted site but also improves its efficacy. Thus, the combination of drugs or moieties with RIT can be applied to overcome the barriers that RIT faces for solid tumors. This review covers the RIT approach, along with the mechanism of action of mAb used in RIT, limitations of solid tumors, and strategies that can be used in combination RIT to enhance the treatment regimen for solid tumors.

## 1. Introduction

Radioimmunotherapy (RIT) in nuclear medicine involves the injection of a radioisotope-labeled monoclonal antibody (mAb), using intravenous or intratumoral injection [1], to treat cancer [2,3,4]. With RIT, radionuclides are carried directly toward the tumor and emit radiation-induced double strand DNA breaks, finally inducing cell death [5,6]. RIT such as ^131^I-tositumomab (Bexxar, GlaxoSmithKline, Philadelphia, PA 19112, USA) [7], ^131^I-rituximab (Rituxan; Genentech, CA, USA) [8], and ^90^Y-ibritumomabtiuxetan (Zevalin; Biogen Idec, Cambridge, MA, USA) [9,10] were administered for treatment of hematological tumors. However, RIT for solid tumor has been less applied in clinics, due to barriers created by tumor microenvironments, such as tumor heterogeneity, abnormal structures of the tumor vessels, highly fibrotic or desmoplastic tumors, absence of functional lymphatics, and high interstitial fluid pressure (IFP) within the tumor [11]. Thus, combination strategies of RIT were investigated for solid tumors. A number of solutions, such as fractionated doses [12], mAb pre-targeting [13,14,15,16], and affibody [17], were investigated to improve the efficacy of RIT. Fractionated RIT using ^90^Y-clivatuzumab was performed to treat patients with stage III/IV pancreatic ductal carcinoma [12]. Regarding mAb pre-targeted strategy, CD38 pre-targeted RIT was performed for B-cell tumors [14]. Another approach to enhance the penetration of mAb was the use of small target protein and then an antibody such as an affibody [17].

In this paper, we review the basic mechanism, limitations, and combination strategies of RIT used to enhance its therapeutic efficacy for solid tumors.

## 2. Isotopes for RIT

**Figure 1:**
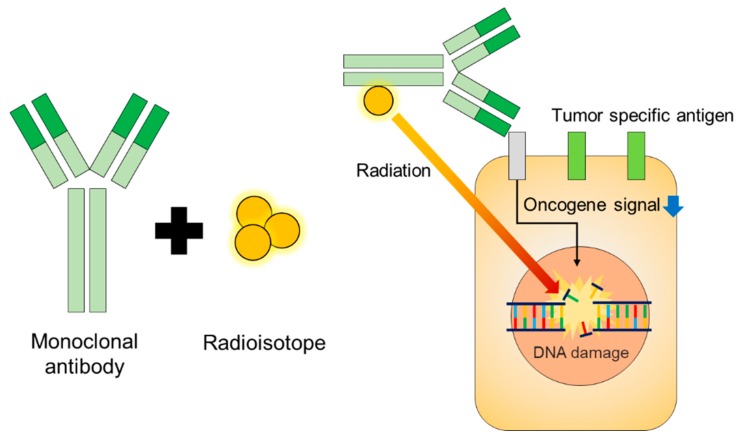
Schematic of radioimmunotherapy (RIT). RIT delivers radiation directly to the tumor-specific antigen of a specific cancer type. The downward blue arrow shows downregulated oncogene signals

Various radioisotopes have been used for RIT. For example, bulky tumors can be treated with radionuclides that emit long-ranged β-rays, such as ^131^I or ^90^Y, whereas small clusters of cells or tumors can be effectively targeted by α-particles [18] or short range β-rays [4]. A schematic of RIT is shown in Figure 1. Characteristics of radionuclides for RIT are shown in Table 1. Examples of commercialized mAbs used in RIT are listed in Table 2.

## 3. Mechanism of Therapeutic Monoclonal Antibodies

Therapeutic mAbs work by targeting specific cell-surface receptors and destroying cancer cells via various mechanisms [19]. Cancer cells use oncogene protein signaling, such as EGFR and HER-2, for survival and proliferation. Therapeutic mAbs are designed to bind with the tumor-specific antigen or oncogene proteins, such as EGFR, Her2, or CD20. For example, Trastuzumab binds to HER2 receptor directly [20] and inhibits the tyrosine kinase signal. This mechanism reduces tumor growth, proliferation, and cell survival signal. Rituximab binds to the CD20 membrane, thereby inducing cellular apoptosis signaling [21].

**Figure 2:**
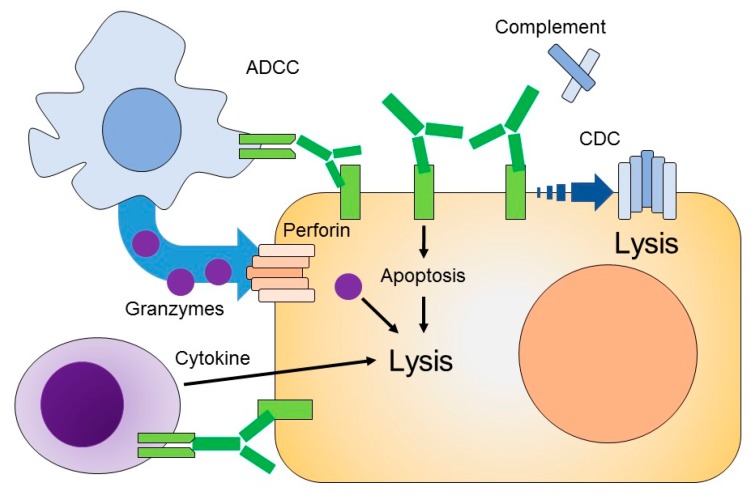
Mechanism of action of an mAb. The mAb binds to its targeted antigen and provokes antibody-dependent cellular cytotoxicity (ADCC) and a complement-dependent cytotoxicity (CDC) response. ADCC includes (natural killer) NK cells, which release granzymes and cytotoxic agents that are responsible for cell lysis. The Fc region of mAb may also bind to soluble protein C1q to promote a cascade reaction that eventually forms a membrane attack complex, as in the CDC-mediated response. Membrane attack complex (MAC) is responsible for disrupting cell membrane and inducing cell lysis.

The mechanism of therapeutic mAb originates from its various functions. A mAb neutralizes the pathophysiological function of the target moiety by binding to the surface-expressed receptor, facilitating antibody-dependent cellular cytotoxicity (ADCC), antibody-dependent phagocytosis (ADCP), and complement-dependent cytotoxicity (CDC) [22]. Figure 2 depicts the mechanism of action of mAb. The mAb binds to the target antigen by its Fv domain, which inhibits the cellular signaling of target cells. Thus, the targeted tumor cell becomes resensitized to cytotoxic agents. Triggering the cytotoxic immune effector cells, like dendritic cells and macrophages, is followed by the process of immune-effector cell lysis of the target cell, as in ADCC or by phagocytosis (ADCP). Similarly, C1 of the CDC process binds to the antibody–antigen complex and initiates a complex process to target cell lysis [23].

### 3.1. Antibody-Dependent Cellular Cytotoxicity (ADCC)

The ADCC and ADCP response is elicited when the immune cell binds to the Fc region of the antibody, while the antibody is targeted to the antigen. In this way, mAb mediates the ADCC response by collaboration with cytotoxic cells like natural killer cells (NK) [24] and macrophages [25]. NK cells bear Fc gamma receptors on their surfaces and release cytotoxic agents, like perforin and granzymes, upon activation. Perforin binds to the target cell’s plasma membrane and creates a pore [26], and granzymes induce apoptosis and fragmentation of cellular DNA [27]. NK cells are activated by binding with the Fc region of immunoglobulins. Apart from releasing the cytotoxic mediator that is responsible for lysis of the tumor cells, NK cells secrete interferon-γ, thus playing a role in the recruitment of adaptive immune cells [28].

### 3.2. Complement-Dependent Cytotoxicity (CDC)

The mAb binds antigens via the Fv region, where the Fc domain may bind to soluble protein complex C1q, leading to the activation of complement cascade that eventually causes cell death. The antigen-targeted mAb and complement complex, such as C5b to C9, are deposited at the target cell′s membrane, forming a cylindriacal membrane attack complex (MAC). MAC disrupts the cell membrane of target cells and is responsible for lysis of tumor cells [28].

## 4. Mechanism of Currently Commercialized mAb for Radioimmunotherapy

### 4.1. Trastuzumab

The transmembrane tyrosine kinase receptors, which include the HER family, regulate cell survival and growth. They are responsible for cell adhesion, migration, and differentiation. Ligand binding generally induces the tyrosine kinase domain, and the receptors are activated by both homodimerization and heterodimerization. Activation may also occur via overexpression of the receptor or by mutation. The overexpression of the HER-2 receptor is mostly observed in breast and gastric cancers. Trastuzumab is the monoclonal antibody composed of two antigen-specific binding sites and rest resembles Fc of immunoglobin G (IgG). Trastuzumab works via several mechanisms, including (1) preventing HER-2 receptor dimerization, (2) destroying the receptor by endocytosis, and (3) inhibiting the shedding of the extracellular domain and immune activation. Trastuzumab mediates the antibody-mediated cell cytotoxicity by recruiting various effector cells [20,29].

### 4.2. Bevacizumab

Vascular endothelial growth factor (VEGF) is a protein that has angiogenic activity. *VEGF* was initially purified from fluid released from a tumor and was named *VEGF-A*. The genes of the VEGF family include *VEGF-A*, *VEGF-B*, *VEGF-C*, and *VEGF-D*, and they are found in mammalian genomes, including those of humans. VEGFR is the tyrosine kinase receptor for *VEGF,* which includes a ligand binding extracellular domain, transmembrane domain, and a cytoplasmic domain. Signaling in typical kinase receptor activates *Ras* (Kristen rat sarcoma) and *PI3K* (Phosphatidylinositol 3-kinase) pathways. *PLCv* (Phospholipase v, *PKC* (Protein kinase C) and *MAPK* (Mitogen activated protein kinase) pathways have also been observed to be activated by *VEGF* bound to *VEGFR-2*. Bevacizumab (anti-VEGF-A humanized monoclonal antibody), commercialized under the name of Avastin^®^, (Genentech, CA, USA) works via anti-VEGF-A/VEGFR therapy. It is approved for colorectal, breast, renal, and small-cell lung cancer and glioblastoma therapy [30]. Clinical data for bevacizumab with other chemotherapeutic agents in the CAIRO2 and PACCE trials also showed lower effectiveness in treatment and survival studies [31,32]. Bevacizumab has been tested in triple negative breast cancer in three steps: (1) pre-targeted approach for fast clearance, (2) low toxicity, and (3) higher therapeutic efficacy [33]. Using an animal model, the researchers stated that in the normal state, the blood vessels remain relatively independent of the *VEGF* level; in contrast, immature tumor vasculature and associated vessels spared by pericytes were observed to disrupt the *VEGF* expression level [34]. However, the reoccurrence of the tumor model in a microscopy study was also observed, and the mechanism was described as follows: continuous anti-VEGF therapy leaves behind the intact basement membrane that serves as a scaffold and enables the rapid regrowth of the vasculature [35]. In addition to the increase in the tumor vasculature, *VEGF* overexpression has been associated with the vascular permeability, which results in an elevated level of interstitial fluid pressure, insufficient blood flow, and poor to uneven perfusion. These factors constitutively hinder drug delivery, especially mAb. The proposition of normalization has gathered research support, where pruning of the immature abnormally functioning vessel sprouts improves delivery of cytotoxic drugs. A possible mechanism explaining this function is the release of a vasodilator mediator, like nitric oxide or prostacyclin, upon downstream signaling generated via *VEGF-2* activation by *VEGF*. Anti-VEGF therapy thus restricts the vasodilators and produces relative vasoconstriction that eventually limits the tumor blood volume. Apart from these mechanisms, another factor affecting apoptosis in tumor cells was examined by using anti-VEGF therapy via *NRP-1* (Neuropilin-1) and *NRP-2 (Neuropilin 2).* VEGF promotes survival signals in breast carcinoma [20,30]. However, the antibody penetration into the tumor with antiangiogenic therapy is controversial. The additive effect of bevacizumab was not observed during radioimmunotherapy [36]. Arjaans et al. showed that blood vessels are normalized via bevacizumab treatment, which is an antiangiogenic drug that does not improves antibody uptake but decreases mean vascular density [37]. Therefore, extensive research is needed to evaluate normalization and drug delivery via anti-angiogenic therapy for clinical use [38].

### 4.3. Cetuximab

Cetuximab works by blocking the epidermal growth factor receptor (EGFR). The *EGFR* is a transmembrane glycoprotein that is composed of an extracellular ligand-binding domain, a transmembrane domain, and an intracellular tyrosine kinase domain. The *EGFR* plays an important role in the growth and sustenance of many human tumors and is expressed on the epithelial surface of the breast, brain, and prostate cancer. This receptor binds to *EGFR* ligands and triggers a cascade of signaling. Cetuximab is approved in irinotecan-intolerant advanced metastatic colorectal cancer and second line therapy with squamous cell carcinoma of the head and neck. Cetuximab antitumor efficacy is categorized into two groups: *EGFR* signal transduction and tumor antigen-targeted cellular immunity. Cetuximab has been shown to be effective in both monotherapy and in combination with other agents and chemotherapeutic drugs, especially for tumors with wild-type *KRAS* activity and metastatic colorectal cancer. Cetuximab efficiently treats metastatic colorectal cancer, but the response-rate values are still insignificant; therefore, targeted therapies are critically important and should be applied to patients with the purpose of optimizing treatment. Markers in *EGFR* that include both clinical and biological are agents for forecasting the response of cetuximab. *KRAS* is the most efficient biomarker, and patients with *KRAS* mutations benefit from cetuximab-based treatment. The responses and reactions of other biomarkers, like *BRAF* (*member of Raf-family (rapidly activated fibrosarcoma*)), *NRAS (Neuroblastoma viral Ras Oncogene homolog)*, *PIK3CA (Phosphotidyl inositol-4,5-Bisphosphate 3-Kinase Catalytic Subunit Alpha)*, and exon 20 are currently being studied [39].

Tumor cells use the immune escape mechanism to elude mAb-induced antitumor response, and cetuximab may provide assistance with differential clinical reactions to tumor antigen (TA)-targeted mAb immunotherapy. In some cases, the combination of TA-specific mAb-based immunotherapy and administration of cytokines or immune adjuvants may benefit patients. EGFR transduction is complex, and tumor cells can avoid effects of cetuximab. Despite hampering EGFR and renewing the ADCC reaction, cetuximab can also restrict articulation of VEGF and cancer angiogenesis. Basic and clinical research should develop cetuximab-based therapies, as existing predicting markers are unsatisfactory [39,40].

### 4.4. Rituximab

Rituximab is essential in the treatment process of malignant tumors, particularly of various B-cell malignancies. In some cases, rituximab alone can produce highly positive reactions and long-term abeyance, whereas, in others, it directly enhances the response rate and overall survival. Despite its role as part of the treatment for tumors, it does not work universally for all patients. Various reactions and instances of resistance have been observed. Without immune-effector mechanisms, rituximab can cause death of malignant cells in vitro; however, this differs from one cell line to another. Rituximab works via (1) direct signaling-induced cell death, (2) complement-mediated cellular cytotoxicity, and (3) antibody-dependent cellular cytotoxicity. Changes that are recognized in the response of rituximab in vitro include hindrance of p-38 mitogen-activated protein kinase, nuclear factor-κB (NF-κB), extracellular signal-regulated kinase 1/2 (ERK 1/2), and *AKT* anti-apoptotic survival signaling pathways, though rituximab is not associated with genetic changes in the CD20 molecule but is connected with changes in signaling. Detection of signaling changes of rituximab response requires cross-linking of rituximab with other antibodies. However, whether it proves useful in clinical therapy has not been determined. Large cell lymphoma and mantle cell lymphoma are less sensitive to rituximab therapy; follicular lymphoma cells are more sensitive. Various studies showed that cell-mediated cytotoxicity (CMC) could aid in the antitumor activity of rituximab in the extravascular and intravascular compartments. CMC can result in quick cell death of rituximab-coated target cells, and is a basic mechanism of action for antibody therapy in some animal models; however, the degree to which it is effective is not yet clear, but the clinical capability is strong enough to promote the development of next-generation CD20. The most promising evidence that ADCC is involved in the clinical response to rituximab therapy was produced from correlative studies illustrating an association between polymorphisms on CD16 and the clinical response to rituximab. Studies point to more than one mechanism playing a role in therapy, including an increase in complement inhibitory molecules, decreased expression of CD20, and enhanced expression of anti-apoptotic molecules. Identification of rituximab mechanisms may not be applicable to all anti-CD20 mAbs, as emerging evidence revealed that not all anti-CD20 mAbs are alike. Clinical trials based on these data include evaluation of antibodies with an enhanced ability to signal and mediate CMC and ADCC. Such findings have to be supported by rigorous correlative analysis, and will add to our understanding of the various mechanisms of action of rituximab in different settings [21].

### 4.5. Immune Suppression Checkpoint Inhibitor

The mAbs that target checkpoint proteins include PD-L1 on tumor cells, PD-1, and CTLA4 on T cells. Examples of PD-1 inhibitors include pembrolizumab (keytruda ^(Rx)^), nivolumab (opdivo ^(Rx)^), and cemiplimab (libtayo). Examples of PD-L1 inhibitor include atezolizumab (tecentriq ^(Rx)^), avelumab (bavencio ^(Rx)^), and durvalumab (Imfinzi ^(Rx)^). An example of the CTLA-4 inhibitors is ipilimumab (yervoy ^(Rx)^) [41]. The immune system maintains immunity via immune surveillance and editing, where T cells continually patrol to find and kill antigens. Several factors are responsible for immune suppression by tumor cells. The most commonly described factors are the loss of tumor antigen and cytotoxicity resistance development through which immune T cells, when near tumor cells, are unable to recognize the antigen. Immune suppression checkpoint inhibitor enhances T cell immunity by modulating the tumor cell and immune cell interaction [42].

## 5. Limitation of RIT for Solid Tumors and Combination RIT Strategies

**Figure 3:**
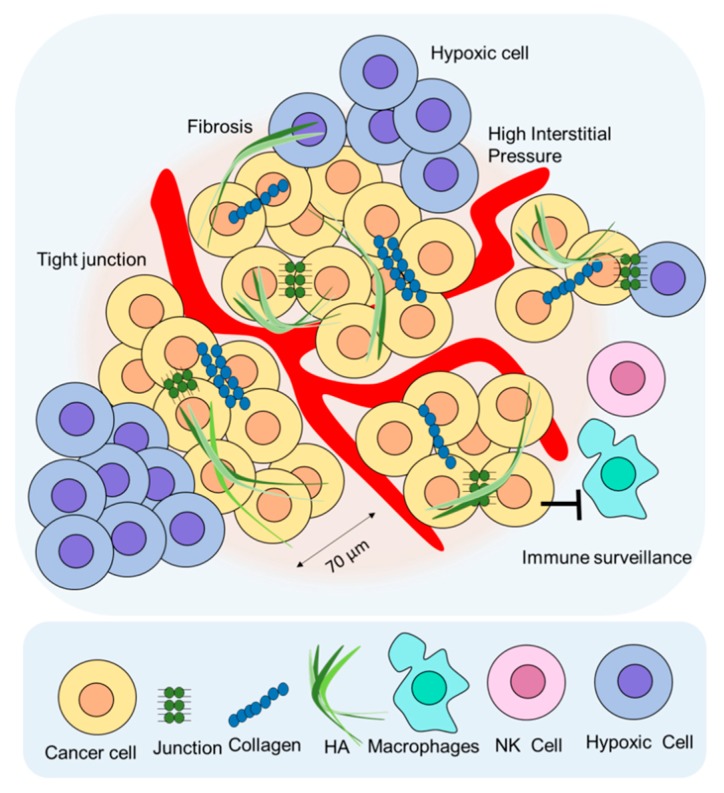
Limitations of RIT in terms of drug delivery. The extracellular matrix (ECM) is composed of collagen, hyaluronic acid (HA), and fibroblasts that hinder drug delivery. Tight junctions and high interstitial pressure are some of the restrictions to RIT delivery in solid tumors.

Theoretically, RIT could be a good candidate for antitumor therapy. However, clinical application of RIT for solid tumors has been limited due to the presence of external and internal barriers during RIT for solid tumors. External barriers include high interstitial pressure [36,43], extracellular matrix (ECM) [44], tight junctions [45], and an immunosuppressive environment [46]. Internal barriers include the modulation of the tumor signal pathways by drugs or medicine targeting the hallmarks of cancer [47]. In this review, we focused on the external barriers to RIT. Figure 3 depicts a schematic of the factors limiting RIT application for solid tumors.

**Figure 4:**
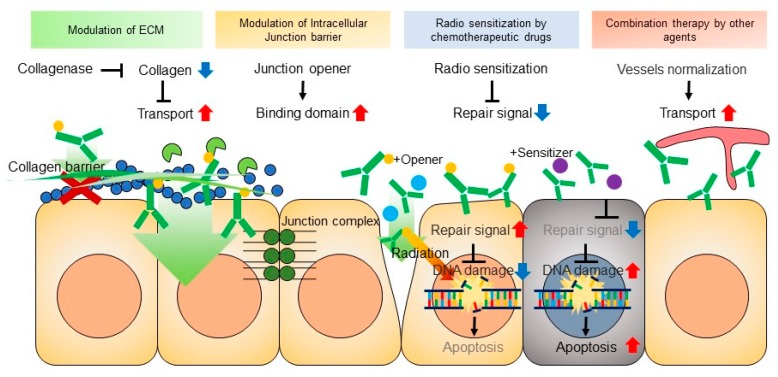
Strategies for combination RIT. The ECM was found to present a considerable barrier to mAb penetration; therefore, ECM-degrading elements are crucial when designing a combinational RIT strategy. Another approach involves liberating tight cell–cell junctions by using drugs that target junctional proteins. Chemotherapeutic agents are cytotoxic agents that are commonly applied in combination RIT. Cell damage reduces the solid stress, which eventually reduces the interstitial pressure and improves RIT efficacy. The combination of RIT with other agents enhances drug transport by vessel normalization or by agents increasing antitumor immunity. The downward blue arrow shows decreased collagen and downregulated oncogene signaling. The upward red arrow shows improve transport and upregulated oncogene signaling.

Various preclinical data about overcoming the limitations to RIT delivery show promise for future clinical trials. In all instances of overcoming the limitations of RIT in solid tumors, RIT demonstrates a promising therapeutic strategy for cancer treatment [48,49]. Herein, we also reviewed the possible strategies for enhancing the effect of RIT for solid tumors. Figure 4 shows existing and potential strategies for RIT.

### 5.1. Extracellular Matrix (ECM)

The tumor-associated ECM is one physical barrier to mAb transport. Glycoproteins and polysaccharides are the major components of the ECM that collectively join to produce the basement membrane [50]. The basement membrane separates the epithelial, endothelial, and interstitial matrix; it is composed of type IV collagen, laminins, and linker proteins, such as nidogens and fibronectin. The interstitial matrix is composed of fibrillary collagens, proteoglycans, and glycoproteins, like tenascin-C and fibronectin, and functions in tissue flexibility. The ECM anchors the basement membrane to maintain tissue polarity. ECM may also be involved in cell migration through the ECM and act as a barrier. The ECM contains components that bind to growth factor receptors, such as heparin sulfate and hyaluronic acid. The ECM can remodel in different tissues, depending upon the context. Another feature of ECM in TME is the cell–ECM interaction facilitating cell rearrangement, realignment, or lysis [44]. Any dysregulation of ECM remodeling enzymes can lead to a disease state and is regarded as a cancer hallmark [46,51]. Highly expressed ECM could increase solid-stress (SS), and this SS interrupts drug transport of mAb and macromolecules [52].

ECM-degrading enzymes have shown promise. In an osteosarcoma model with highly expressed collagen, drug transport was improved by collagen-degrading enzyme collagenase [53]. Enhanced penetration of mAb in human osteosarcoma xenografts was achieved after the injection of collagenase [53]. Another ECM component, hyaluronan, was reported to promote cell progression and restrict the binding of trastuzumab to its receptor [54]. By developing drugs that inhibit hyaluronan, trastuzumab binding to its receptor can be enabled. The use of carriers, such as hydrogel-based drug delivery with collagenase and trastuzumab localized delivery, have shown therapeutic potential for drug penetration by the degradation of the ECM [55]. ECM remodeling by the oncolytic vaccinia virus enhanced the efficacy of immune-checkpoint blockade [56]

### 5.2. Cell-to-Cell Junctions

Cell-to-cell junctions regulate the permeability of the two layers of cells and provide a method of communication between cells and the plasma membrane [57]. Tight junctions are the guard of paracellular pathways. However, cell-to-cell junctions in cancer reduce drug transport [53]. Epithelial junctions reduce transport of trastuzumab and cetuximab in solid tumors [58]. Tight junctions reduce the killing effect by trastuzumab [59]. Wang et al. showed that reducing the tight junction via adenovirus serotype 3 could increase the anticancer effect of trastuzumab in a breast-cancer xenograft model [59]. Similarly, desmoglein-2, an epithelial protein, served as the primary attachment receptor for human adenovirus [58]. The authors derived a protein from human adenovirus that targeted desmoglein-2 and named it junctional opener-1 (JO-1). JO-1 for mAb was effective in the treatment of a solid tumor.

### 5.3. High Interstitial Pressure

Solid tumors are composed of cancer cells tightly packed together with abrupt and leaky blood vessels and lymphatic vessels. The leaky nature of blood vessels and the drainage of lymphatic vessels increase the interstitial pressure in tumors. Solid tumors have high interstitial fluid pressure (IFP) [60]. Tumor cells generate solid stress, and this stress compresses the surrounding blood vessel and increases the IFP and microvascular pressure [61]. This increase creates a barrier to drug transport. Due to the increase in interstitial pressure, the uptake of therapeutic agents is limited [36,43,62]. The mechanism underlying high IFP is not clearly understood, but various factors are thought to be involved, including the permeable environment of the vessels, irregularities associated with lymph vessels, and interstitial space confined by the fibroblast of the stroma [60]. Human pancreatic cancer orthotropic xenograft mice had higher IFP than in a normal pancreatic tumor group [63]. Solid tumors were found to have a high IFP [64]. Both hydrostatic and osmotic pressure increase in solid tumors [65,66]. The molecular weight of anticancer drugs or proteins are relatively large compared to those of glucose or oxygen. The uptake of anticancer drugs into the tumor area is highly intermittent due to elevated IFP [60]. Many attempts were made to overcome this barrier. High-intensity focused ultrasound (HIFU) provided enhanced uptake of drug by disruption of the stroma [67]. Combination of RIT with HIFU showed higher penetration of mAb [68]. The combination of RIT with paclitaxel also produced enhanced accumulation of mAb in tumor tissue [36]. Tanexe induced apoptosis via disruption of the microtubular network associated with the reduction in interstitial pressure [69].

### 5.4. Immune Surveillance

Interactions between immune cells and tumor cells produced an immunosuppressive environment, wherein the host’s own immune cells were unable to fight the cancer; rather, they participated in growth signaling, promotion, and differentiation. Immunotherapies emphasize the importance of exploiting the interactions of immune cells to kill the cancer cells [70]. PD-1 is an immune inhibitory receptor that is expressed in immune cells like T and B cells; however, cancer cells manage to escape from the immune system using checkpoint proteins such as PD-1/PD-L1 or CTLA-4. These checkpoint proteins have the ability to cheat immune cells [71]. Other authors studied the various mechanisms through which tumor cells suppress the immune system via cell signaling pathway interruption, alter the protein structure that affects regulatory function, or release cytokines and chemokines from various immune cells [72]. These chemokines and checkpoint proteins play a role in cancer progression by altering costimulatory signals and causing a disproportional ratio of regulatory T cell (Treg) or suppressor T cells (Ts) to effector cells, resulting in impaired tumor-specific immune responses [70]. Many proto-oncogenes function to control and regulate various cellular processes. Mutations in any of these could disrupt signaling cascade pathways, which may lead to tumor cells potentially affecting the cellular machinery and creating immune surveillance [73].

Investigations into immune checkpoint inhibitors represent a new horizon in anticancer treatment, with James p. Allison and Tasuku Honju winning the 2018 Nobel Prize in medicine/physiology for their discovery of cancer therapy to inhibit negative immune regulation. Checkpoint inhibitor targets PD-1/PD-L1 or CTLA-4 to overcome an immunosuppressive environment [71]. Although immune checkpoint inhibitors are used as a powerful anticancer therapeutic tool, a PD-1/PD-L1- or CTLA-4-negative patient could not achieve good results by using a checkpoint inhibitor. Abdul et al. showed that blocking PD-L1 combined with irradiation increased antitumor efficacy in pancreatic ductal adenocarcinoma; radiation with anti-PD-L1-treated mice dramatically decreased tumor size and increased immunity [74]. Demaria showed that tumors that are nonresponsive to immune checkpoint inhibitors become responsive after radiation therapy, suggesting radiotherapy sensitizes the immunotherapy. For tumors that express high levels of PDL-1, higher doses of radiation may kill these cells [75].

Although no report has been published on the levels of PD-1, PD-L1, or CTLA-4 during RIT, the effect should be investigated, since RIT carries radiation in combination with mAb.

### 5.5. Hypoxia

Cancer cells are crowded and compete with normal cells for the supply of oxygen. A limited supply of oxygen to the cancer cell drops the physiological oxygen level and creates a hypoxic microenvironment for tumor cells, the severity of which depends upon the tumor type.

Theoretically, the supply of oxygen is about 70 μm to less than 90 μm from the disrupted vessel produced in the tumor microenvironment. Any area beyond that, approximately 100 μm from the vessel or with no direct contact with capillaries, is considered hypoxic. Hypoxia is one of the challenging limitations in terms of the radiation treatment. Molecular oxygen is a strong radio sensitizer that captures electrons through the absorption of energy from the radiation source, and O^2^ plays a part in the DNA damage process [76,77]. In terms of treatment concerning radiation, the nature of the oxygen mechanism is explained by the oxygen enhancement ratio (OER). The OER is the ratio of radiation doses of hypoxic to aerated conditions that produces the same biological effect. It is thought that the oxygen effect is largely intermediately observed under sparsely ionizing radiation, like X-rays used in external beam therapy with β-radiation, but for severely ionizing radiation like α-particle-based radiation therapy, the oxygen effect is negligible. Hence, RIT composed of α-particle-based radiation antibody therapy is promising. However, the limitation of α-particle-based radiation is its short range that cannot reach hypoxic regions. So, hypoxia generates radiation resistance due to reduced therapeutic response, which is why improving mAb penetration toward the tumor core, deeper into the tumor tissue, is important.

To combat the hypoxic changes, cancer cells alter their metabolism, [33] promote angiogenesis [78], and acquire mobility and metastasis by transitions from an epithelial to mesenchymal phenotype [79]. *Hypoxia inducible factor* (*HIF*) is a transcription gene that stabilizes under hypoxia but degrades under a normoxic environment. The degradation of HIF-α is regulated by Von Hippel–Lindau tumor suppressor (*pVHL*). Structurally, the HIF is a heterodimer, comprised of *HIF-1α* and *HIF-2α* subunits, and regulated by oxygen concentration. Both subunits share high identity in their functional domain; however, the expression levels are highly variable. The transcriptional gene *HIF-1α* plays a responsive role at low oxygen levels, controls vascularization and tumor growth by interacting with enzymes and other transcriptional factors, and is ubiquitously expressed in mammalian cells and tissue. *HIF-2α* responds at higher oxygen concentrations and for a longer duration, and it is abundant in endothelial and vascularized cells. Since hypoxia offers resistance to radiotherapy, HIF-1 is considered a therapeutic target in cancer therapeutics [80,81].

### 5.6. Combination of RIT with Other Agents

Combination RIT is the approach where any substance, drug, and moiety that has the potential to either cause apoptosis, decrease interstitial pressure, degrade the ECM, overcome the junctional barrier, overcome immunosuppression or act as a radiosensitizer is used, along with RIT, to enhance RIT delivery and to improve therapeutic efficacy.

A radiosensitizer is a molecule, moiety, or drug that increases the sensitivity of cancer cells to radiotherapy and is nontoxic to normal cells at therapeutic doses [82]. Ng et al. reported tumor regression and prolonged tumor-free survival when the topoisomerase-inhibition drug, topotecan, was used in combination with RIT in a xenograft model of breast cancer [83]. Milenic et al. reported the potentiation effect of high linear energy transfer radiation with ^212^Pb and trastuzumab combined with gemcitabine, which resulted in a median survival of 66 days compared with 34 days without gemcitabine treatment. The study was conducted in xenograft mice bearing LS-174T cells, to study disseminated peritoneal disease [84]. The combined effect of chemotherapy drugs and RIT on gene expression levels was also studied. Yong et al. explored an alkaloid chemotherapeutic, paclitaxel, along with α-emitting ^212^Pb radiation combined with mAb for HER2, and quantified 84 DNA damage response genes. They reported the killing effect of combination therapy by genes linked to the mitotic spindle checkpoint and BRACA1-(Breast Cancer Gene1) associated genome surveillance complex [85]. In support of combination therapy with paclitaxel as a radiosensitizer, Cividalli et al. explored the enhanced efficacy of paclitaxel in vivo with different doses, reporting a synergistic effect [86]. Apart from combining α-emitting radiation, paclitaxel was combined with a β-emitting, radiolabeled, humanized mAb in a breast cancer model. Significant potential was observed with combined modality treatment, as explored by Kelly et al. [87]. Kurizaki et al. [88] combined ^125^I-B72.3 with a selective peptide agonist of human C5a (GCGYSFKPMPLaR) (AP), to enhance the efficacy of RIT. Parecoxib (cyclooxygenase-2 inhibitor), as a radiosensitizing agent in combination with RIT, was also reported [89]. The antitumor response with celecoxib, a selective Cox-2 inhibitor, and agonistic anti-CD40 were also used for combinational immunotherapy during treatment of malignant glioma patients and is thus a candidate for RIT [90].

### 5.7. Improving Pharmacokinetics of mAb with Pre-Targeting

A critical challenge in the therapeutic use of mAb is its slow pharmacokinetics characteristic. The size of intact mAb is ~150 kDa, which is larger than other molecules used in nuclear medicine. Since the circulatory residence time is long, which is beneficial because the labeled or unlabeled targeted moiety is in circulation for a longer time, enabling the delivery of the desired therapeutic efficacy. However, the high risk of a cross-firing effect exists if the mAb is labeled with a radioactive radionuclide, particularly in the case of long-range β-emitters like ^131^I or ^90^Y. Pharmacokinetics values can be improved by pre-targeting [91]. The idea behind this is that the labeled antibodies, in intact or in fragmented form to reduce their molecular size, or in the form of recombinant, function to carry bounded radionuclides to the tumor site. The multistep pre-targeting radio immunotherapy method first delivers antibodies by using the streptavidin method or biotin-avidin method, or by the use of bispecific antibodies, thus allowing considerable time for the excess mAb to eliminate. This is followed by the radiolabeled heptane to be directed toward the mAbs. As such, the time for the radioactivity to spread toxicity is constrained, becoming more efficacious in therapeutic levels. Bispecific haptens engineering allows cooperative binding, so two different antigen scan be targeted by incorporating two different antibodies. Examples of pre-targeted RIT in reported studies are listed in Table 3.

## 6. Conclusions

RIT for solid tumors may be efficacious after modulation of barriers. Therefore, strategies and approaches should be implemented to achieve RIT application in clinical studies. Evidence from both animal and clinical studies have highlighted the importance of combination RIT, as it can overcome various internal and external barriers and clear the pathway for mAb penetration into solid tumors, thus improving the efficacy of conventional RIT.

## Figures and Tables

**Table 1:** Radionuclides for RIT.

Radioisotopes	Max Range (in Water)	Half-Life	Max Energy (keV)
*β*-emitter			
^67^Cu	2.1 mm	61.9 h	575
^90^Y	11.3 mm	64.1 h	2284
^131^I	2.3 mm	8.0 days	606
^177^Lu	1.8 mm	6.7 days	497
*α*-emitter			
^211^At	<50 μm	7.2 h	586
^213^Bi	<50 μm	45.6 min	5870
^225^Ac	<50 μm	240 h	5830
^223^Ra	<100 μm	11.4 days	5979

**Table 2:** Monoclonal antibodies and their tumor-specific antigen used for RIT.

Monoclonal Antibody	Targeted Site
Trastuzumab (Herceptin, Roche, Basel, Switzerland)	HER2
Bevacizumab (Avastin, Genentech, CA, USA)	VEGF-A
Cetuximab (Erbitux, Kenilworth, NJ, USA)	EGFR
Rituximab (Rituxan, Genentech, CA, USA)	CD20
Tositumomab (Bexxar, Genentech, CA, USA)	CD20
Ibritumomab tiuxetan (Zevalin, Biogen Idec, Cambridge, MA, USA)	CD20

**Table 3:** Examples of studies of pre-targeting RIT alone and in combination therapy with pre-targeted RIT studies.

Aim	Radionuclide	Cancer Type	Targeted Antigen	Subject	Combination	Reference
To determine maximum tolerated dose and antitumor efficacy of three-step pre-targeting method	^90^Y	Glioma	Tenascin	Human	No	[92]
Safe and effective with negligible toxicity pre-targeting RIT	^90^Y	LS-180, human carcinoma	Ep-CAM	BALB/c nude mice	No	[93]
Response to combination pre-targeted with less toxicity	^90^Y	LS174T, human colon adenocarcinoma	Tag-72 (CC49)	BALB/c nude mice	Gemcitabine	[94]

## Abbreviations

RIT	Radioimmunotherapy
mAb	Monoclonal Antibody
ADCC	Antibody-dependent cellular cytotoxicity
ADCP	Antibody-dependent cellular phagocytosis
MAC	Membrane attack complex
CDC	Complement-dependent cytotoxicity
PI3K	Phosphatidylinositol 3-kinase
PLCv	Phospholipase v
PKC	Protein kinase C
MAPK	Mitogen activated protein kinase
KRAS	Kristen rat sarcoma
CMC	Cell-mediated cytotoxicity
